# Prevalence and factors associated with sarcopenia in individuals with stroke undergoing rehabilitation: A cross-sectional study

**DOI:** 10.1371/journal.pone.0361113

**Published:** 2026-10-26

**Authors:** Fernando Shizuo Ida, Esther Joyce De Monte Castello E. Silva, Gabriela De Souza Martins Brasil, Leandro Moreira Peregrino, Cruiff Emerson Pinto Da Silva, Pedro Ykaro Fialho Silva

**Affiliations:** 1 SARAH Network of Rehabilitation Hospitals, Fortaleza, Ceará, Brazil; 2 Graduate Program in Physiotherapy, Federal University of Ceará, Fortaleza, Ceará, Brazil; 3 Graduate Program in Public Health, Federal University of Ceará, Fortaleza, Ceará, Brazil; Mongolian National University of Medical Sciences, MONGOLIA

## Abstract

**Objective:**

To determine the prevalence of sarcopenia and identify clinical and functional factors independently associated with confirmed sarcopenia in individuals with stroke undergoing rehabilitation.

**Methods:**

This cross-sectional study enrolled 209 adults with stroke admitted for neurological rehabilitation. Sarcopenia was defined according to the European Working Group on Sarcopenia in Older People 2 (EWGSOP2) algorithm, using SARC-CalF, handgrip strength, five-times sit-to-stand test, physical performance measures and appendicular skeletal muscle mass by dual-energy X-ray absorptiometry (DXA). Bivariate analyses and multivariable logistic regression were performed to identify factors independently associated with confirmed sarcopenia.

**Results:**

Confirmed sarcopenia was identified in 55 of 209 participants (26.3%; 95% CI: 20.6% to 32.8%). Participants with sarcopenia were older and had lower body mass index (BMI), poorer physical performance, and greater motor impairment than those without sarcopenia. In the multivariable model, lower BMI (OR = 0.70; 95% CI: 0.59 to 0.83), positive SARC-CalF screening (OR = 6.37; 95% CI: 2.03 to 20.05), and lower Short Physical Performance Battery (SPPB) scores (OR = 0.83; 95% CI: 0.69 to 0.99) remained independently associated with sarcopenia. Motor impairment severity, assessed using Fugl-Meyer motor categories, was also independently associated with sarcopenia.

**Conclusion:**

DXA-confirmed post-stroke sarcopenia affected approximately one in four individuals undergoing rehabilitation and was independently associated with lower BMI, poorer physical performance, and greater motor impairment. These findings indicate that sarcopenia is closely linked to functional impairment after stroke and reinforce the importance of integrating muscle health and functional assessment during rehabilitation. Early identification of individuals at risk may support more individualized rehabilitation strategies and improve clinical decision-making.

## Introduction

Sarcopenia is a progressive musculoskeletal disorder characterised by loss of skeletal muscle mass, strength, and physical performance, and is associated with adverse outcomes, including disability, falls, prolonged hospitalisation, and mortality [1]. In the context of stroke, sarcopenia is of particular clinical relevance, as the interaction between central neurological injury and peripheral musculoskeletal changes may adversely affect functional recovery [2,3].

The burden of post-stroke sarcopenia appears substantial. Systematic reviews and meta-analyses have reported prevalence estimates ranging from approximately 37% to 42%, although these vary depending on diagnostic criteria, stroke phase, and study population [4,5]. Despite this, sarcopenia remains under-recognised in neurorehabilitation, partly due to its diagnostic complexity and the limited integration of muscle assessment into routine clinical practice [1,5]. This gap is clinically important, as sarcopenia represents a potentially modifiable contributor to post-stroke disability.

The pathophysiology of post-stroke sarcopenia is multifactorial. Neurological deficits impair motor unit recruitment and reduce physical activity, accelerating muscle loss, particularly in the paretic limb. In parallel, systemic factors such as inflammation, metabolic dysregulation, and inadequate nutritional intake further contribute to muscle wasting [6,7]. These mechanisms highlight the complex interplay between neurological impairment and muscle dysfunction.

Clinically, sarcopenia has been associated with poorer functional outcomes, reduced independence in activities of daily living, and impaired mobility [8]. Importantly, recovery after stroke is not determined solely by the extent of brain injury. Peripheral factors, particularly muscle strength and quality, play a key role, especially in the subacute and chronic phases when neurological recovery tends to plateau [9,10].

Interventions targeting muscle function, particularly resistance training, have demonstrated meaningful improvements in strength and functional performance, even in later stages of recovery [11]. However, factors associated with post-stroke sarcopenia remain incompletely understood, particularly within rehabilitation settings where early identification of high-risk individuals may inform clinical decision-making [12].

Therefore, this study aimed to determine the prevalence of sarcopenia and identify clinical and functional factors independently associated with confirmed sarcopenia in individuals undergoing neurorehabilitation after stroke.

## Methods

### Study design and setting

This cross-sectional observational study was conducted at a specialised neurorehabilitation centre between 20 January 2025 and 10 August 2025. The study followed the Strengthening the Reporting of Observational Studies in Epidemiology (STROBE) statement for cross-sectional studies [13]. This study was approved by the institutional research ethics committee (approval number: 76013823.7.0000.0022) and conducted in accordance with the Declaration of Helsinki. All participants or their legal representatives provided written informed consent.

### Participants

Adults (≥18 years) admitted for inpatient neurological rehabilitation after ischaemic or haemorrhagic stroke were screened for eligibility. Inclusion criteria were: confirmed diagnosis of stroke based on clinical and neuroimaging findings; classification as subacute (7 days to 6 months) or chronic (>6 months) after stroke; medical stability to participate in assessments; and ability to provide informed consent, or availability of a legal representative. All participants were assessed within 7 days of admission.

Exclusion criteria were: active malignancy or recent chemotherapy/radiotherapy; contraindication to DXA scanning; severe medical comorbidity preventing assessment.

Participants were recruited consecutively to minimise selection bias. All assessments were performed by trained assessors following predefined protocols, and potential confounders, including age, sex, stroke characteristics, and time since stroke, were recorded.

### Sarcopenia assessment

Sarcopenia was defined according to the EWGSOP2 algorithm [1]. Screening was performed using SARC-CalF. Muscle strength was assessed by bilateral handgrip strength (HGS) using a calibrated hydraulic dynamometer (Jamar, Patterson Medical, USA), with the maximum value from either hand retained for classification. Low handgrip strength was defined as <27 kg in men and <16 kg in women. Muscle strength was additionally assessed using the five-times sit-to-stand test (5STS), with values >15 seconds considered indicative of reduced performance.

Physical performance was assessed with the Short Physical Performance Battery (SPPB). Low physical performance was defined as an SPPB score ≤8.

Appendicular skeletal muscle mass was quantified by whole-body dual-energy X-ray absorptiometry (Lunar iDXA, GE Healthcare, USA). According to the study protocol, all attending physicians were instructed to request DXA for every patient with stroke who met the study eligibility criteria upon admission to the rehabilitation program, regardless of clinical suspicion of sarcopenia. DXA examinations were performed by a trained radiology technician under the supervision of a board-certified radiologist. Daily quality control and weekly calibration procedures were performed before patient assessments using the manufacturer’s phantom, according to the manufacturer’s recommendations. All examinations followed a standardised patient positioning protocol, and both professionals were blinded to the study objectives and participants’ clinical and functional assessments. Appendicular skeletal muscle mass index (ASMI) was calculated as appendicular lean mass divided by height squared (kg/m²). Low muscle mass was defined as ASMI <7.0 kg/m² in men and <5.5 kg/m² in women.

According to the EWGSOP2 framework, sarcopenia was identified using a hierarchical, stepwise approach. Case-finding was initially performed with SARC-CalF to detect individuals at risk. Probable sarcopenia was defined by the presence of low muscle strength, primarily determined by reduced HGS, while the 5STS was incorporated as a complementary functional measure to capture impaired lower-limb muscle performance. Confirmed sarcopenia required the coexistence of low muscle strength and low muscle mass (ASMI below the established cut-points), and severe sarcopenia was defined by the additional presence of low physical performance (SPPB ≤8).

### Clinical and functional variables

Demographic data included age, sex, race/ethnicity, education, place of origin, and time since stroke. Clinical variables included stroke type, TOAST classification for ischaemic stroke, comorbidities and associated diagnosis. Anthropometric measures included body mass index (BMI) and calf circumference.

Global disability was assessed using the Modified Rankin Scale (mRS), an ordinal scale ranging from 0 (no symptoms) to 6 (death), with higher scores indicating greater disability [14]. The mRS is widely used and validated for assessing functional outcomes after stroke [15].

Motor impairment was assessed using the motor domain of the Fugl-Meyer Assessment (FMA) for the upper and lower extremities (0–66 and 0–34, respectively), with higher scores indicating better motor function. The total motor score (0–100) was calculated as the sum of both subscales [16].

Walking ability was evaluated using the Functional Ambulation Classification (FAC), a six-level ordinal scale (0–5) that classifies patients according to the level of physical assistance required for ambulation, with higher scores indicating greater independence. The FAC has demonstrated good reliability and validity in stroke patients [17].

Functional independence was assessed using the Functional Independence Measure (FIM), an 18-item scale comprising motor and cognitive domains. Total scores range from 18 to 126, with higher scores indicating greater independence [18].

According to the institutional assessment protocol, the FMA and FIM were not administered to participants with severe cognitive and motor impairments when reliable standardised assessment was not feasible.

### Sample size

Sample size was estimated using the StatCalc module of Epi Info™ 7, assuming a 95% confidence level, 80% power, and an estimated prevalence of 42%, based on the study by Su et al. (2020). Using Fleiss’ method, the minimum required sample size was estimated at 102 participants. During the study period, all eligible patients admitted for rehabilitation were invited to participate, resulting in a final sample that exceeded the minimum calculated size.

### Statistical analysis

Statistical analyses were performed using IBM SPSS Statistics. Descriptive statistics were calculated for all variables. Continuous variables were expressed as mean ± standard deviation or median (interquartile range), according to data distribution assessed by the Shapiro–Wilk test and visual inspection of histograms and Q–Q plots. Categorical variables were presented as frequencies and percentages.

The prevalence of sarcopenia and its stages was estimated as proportions, with corresponding 95% confidence intervals (95% CI) calculated using the exact binomial (Clopper–Pearson) method.

Between-group comparisons (participants with and without sarcopenia) were conducted using independent-samples t-tests or Mann–Whitney U tests for continuous variables, and chi-square tests or Fisher’s exact tests for categorical variables, as appropriate.

To identify factors independently associated with sarcopenia, multivariable logistic regression analysis was performed using a complete-case approach, including only participants with complete data for all variables entered into the final model. Candidate variables were selected based on clinical relevance and statistical significance in bivariate analyses (p < 0.10). Continuous variables were retained in their original scale whenever possible to preserve statistical power. Fugl-Meyer scores were entered as categorical variables according to predefined clinical categories. A backward stepwise selection procedure was applied to derive the final multivariable model.

Multicollinearity was assessed using variance inflation factors (VIF), with values >5 indicating potential collinearity. The assumption of linearity in the logit for continuous variables was evaluated prior to model inclusion. Model fit was assessed using the Hosmer–Lemeshow goodness-of-fit test and model discrimination by the area under the receiver operating characteristic (ROC) curve.

Results are reported as odds ratios (OR) with 95% confidence intervals (CI). Statistical significance was set at p < 0.05 (two-tailed).

Missing data were handled using a complete-case analysis approach. No imputation procedures were performed. Among participants included in the analyses, missing data due to incomplete assessments were infrequent (<3% for all variables). Separately, the FIM and FMA were not administered to participants with severe cognitive and motor impairments when reliable standardised assessment was not feasible, according to the institutional assessment protocol. These cases were reported descriptively and analyses involving these measures were restricted to participants with available assessments.

## Results

### Participant flow

Participant selection process is presented in Fig 1. During the study period, 311 eligible patients were identified. Of these, 209 were included in the final analysis. A total of 102 patients were excluded because DXA examination was not requested by the physician (n = 85), the evaluation protocol was not applied completely (n = 10), or the participant did not attend the DXA examination (n = 7). Preliminary analyses showed no significant differences in age or mRS scores between included and excluded patients. Although physicians were instructed to request DXA for every eligible participant with stroke according to the institutional assessment protocol, DXA was not requested for 85 participants because the protocol was not consistently implemented during routine clinical practice. Consequently, these participants could not be classified according to DXA-confirmed sarcopenia criteria and were excluded from the final analysis.

**Fig 1:**
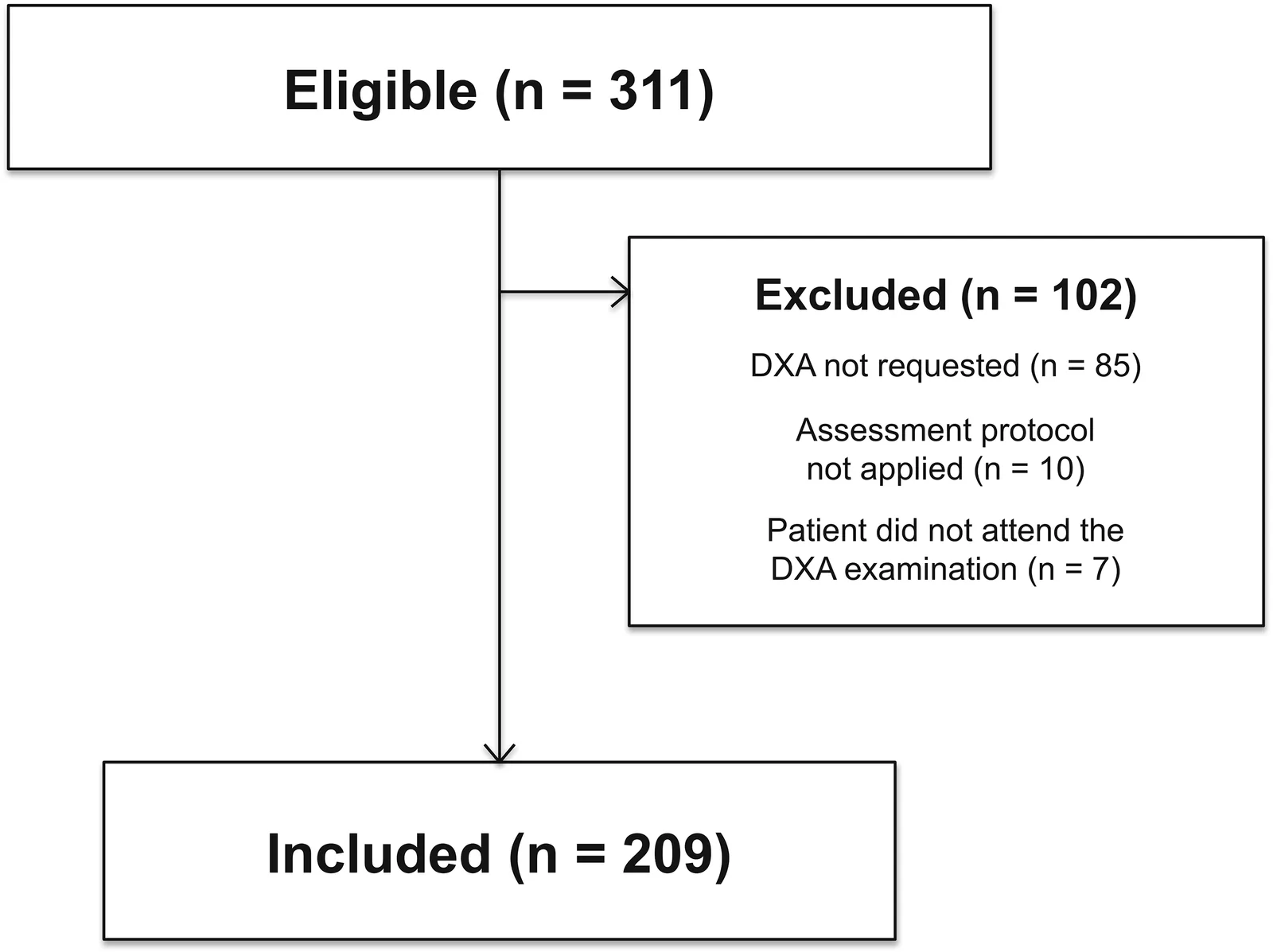
Flow diagram of participant inclusion and exclusion. This figure shows the number of participants assessed for eligibility, excluded, and included in the final analysis.

### Sample characteristics

Baseline sociodemographic, clinical, and functional characteristics of the participants are presented in Table 1. The mean age of participants was 60.6 ± 13.9 years, and 44% were aged 61–75 years. Most participants were male (63.2%), and 64.6% self-identified as mixed race. Stroke was ischaemic in 173 participants (82.8%) and haemorrhagic in 36 (17.2%). Among ischaemic strokes (n = 173), the most frequent TOAST subtypes were large-artery atherosclerosis (35.3%) and cardioembolism (24.9%). Most participants were in the chronic phase after stroke.

**Table 1:** Sociodemographic, clinical and functional characteristics of participants (n = 209).

Variable	n (%)
**Age group (years)**	
18–30	8 (3.8)
31–45	25 (12.0)
46–60	58 (27.8)
61–75	93 (44.5)
≥76	25 (12.0)
**Sex**	
Female	77 (36.8)
Male	132 (63.2)
**Stroke type**	
Ischaemic	173 (82.8)
Haemorrhagic	36 (17.2)
**Time since stroke**	
≤90 days	12 (5.7)
91–180 days	51 (24.4)
181–365 days	49 (23.4)
1–2 years	47 (22.5)
≥2 years	50 (23.9)
**BMI category**	
Underweight	18 (8.6)
Normal weight	79 (37.8)
Overweight	58 (27.8)
Obesity	54 (25.8)
**mRS**	
1–2	66 (31.5)
3	40 (19.1)
4–5	103 (49.3)
**FAC**	
0–2 (non-functional)	106 (50.7)
3 (dependent)	40 (19.1)
4–5 (independent)	63 (30.1)
**FMA (n = 196)**	
Severe	69 (35.2)
Moderately severe	23 (11.7)
Moderate	40 (20.4)
Mild	64 (32.7)
**FIM (n = 183)**	
High dependence	15 (8.2)
Moderate dependence	90 (49.2)
Low dependence	78 (42.6)

BMI = body mass index; mRS = Modified Rankin Scale; FAC = Functional Ambulation Classification; FMA = Fugl-Meyer Assessment; FIM = Functional Independence Measure.

Hypertension was the most common comorbidity (77%), followed by prediabetes/diabetes (52%) and dyslipidaemia (50%). Overweight and obesity together were present in 53% of the sample at admission.

According to the mRS, 40% of participants had moderately severe disability. Twenty-six per cent were non-functional ambulators, and 44% required assistance or supervision for walking. FMA motor impairment severity was available for 196 participants; among those assessed, 67% were classified as having moderate to severe motor deficits. FIM data were available for 183 participants. According to the FIM classification, 49.2% were classified as having moderate dependence and 42.6% as having low dependence.

### Prevalence of sarcopenia

The prevalence of sarcopenia risk, as assessed by SARC-CalF, was 44.3% (90/203; 95% CI 37.4–51.4). Probable sarcopenia was identified in 62.8% of participants based on HGS (130/207; 95% CI 55.9–69.3) and in 78.9% using the 5STS (165/209; 95% CI 72.7–84.2). Confirmed sarcopenia, defined by DXA, was observed in 26.3% of participants (55/209; 95% CI 20.6–32.8), while low physical performance, defined as an SPPB score ≤8, was observed in 80.6% of participants (166/206; 95% CI: 74.5%–85.8%).

### Comparison between participants with and without sarcopenia confirmed by DXA

Table 2 compares participants with and without DXA-confirmed sarcopenia. Participants with sarcopenia confirmed by DXA were significantly older (p = 0.007) and had lower BMI (p < 0.001), lower FMA motor scores (total, upper limb, and lower limb; all p ≤ 0.01), reduced calf circumference (p < 0.001), higher SARC-CalF scores (p < 0.001), lower HGS, and worse performance in the six-minute walk test, SPPB, and FIM (all p < 0.05). No significant differences or associations with confirmed sarcopenia by DXA were observed for sex and race/ethnicity, clinical variables, including stroke type, comorbidities, associated diagnoses, and time since stroke, or functional variables, including mRS, gait speed, 5STS, Timed Up and Go, and FAC (all p > 0.05).

**Table 2:** Comparison of clinical and functional characteristics between participants with and without sarcopenia.

Variable	N	No sarcopenia Mean (SD)	Sarcopenia Mean (SD)	Mean difference (95% CI)	p-value
Age (years)	209	59.1 (14.1)	64.9 (12.5)	−5.8 (−9.8 to −1.8)	0.007
BMI (kg/m²)	209	29.1 (4.8)	23.3 (3.5)	5.8 (4.5 to 7.2)	<0.001
FMA-UL (0–66)	196	36.8 (22.5)	27.5 (21.9)	9.3 (2.2 to 16.4)	0.011
FMA-LL (0–34)	196	23.5 (8.6)	20.2 (9.5)	3.3 (0.5 to 6.1)	0.019
FMA-motor score (0–100)	196	60.3 (29.7)	47.4 (30.2)	12.8 (3.4 to 22.3)	0.008
Calf circumference – right (cm)	209	35.3 (3.6)	30.7 (4.0)	4.6 (3.4 to 5.8)	<0.001
Calf circumference – left (cm)	209	35.4 (3.6)	31.1 (3.3)	4.3 (3.2 to 5.3)	<0.001
SARC-CalF score	203	3.9 (5.2)	9.0 (4.3)	−5.1 (−6.6 to −3.5)	<0.001
HGS (kg)	207	22.2 (9.8)	17.6 (9.4)	4.5 (1.1 to 8.0)	0.011
6MWT (m)	209	276.2 (144.8)	201.0 (135.1)	75.2 (1.5 to 148.9)	0.046
SPPB (points)	206	4.9 (4.1)	2.6 (3.1)	2.3 (1.2 to 3.5)	<0.001
FIM-total	183	77.9 (21.0)	64.5 (22.5)	13.4 (6.5 to 20.4)	<0.001

Values are presented as mean (SD)**.** BMI: body mass index; FMA-UL: Fugl-Meyer Assessment upper limb; FMA-LL: Fugl-Meyer Assessment lower limb; FMA motor score: Fugl-Meyer Assessment total motor score; SARC-CalF: Strength, Assistance with walking, Rise from a chair, Climb stairs, Falls and calf circumference; HGS: handgrip strength; 6MWT: 6-minute walk test; SPPB: Short Physical Performance Battery; FIM: Functional Independence Measure.

### Factors independently associated with sarcopenia

BMI showed a moderate negative correlation with confirmed sarcopenia, while SARC-CalF showed a moderate positive correlation. Weaker correlations were observed for SPPB, FIM, FMA motor score, and 6MWT.

Multivariable logistic regression was performed using variables with p < 0.10 in bivariate analyses and clinically relevant covariates. The final model, obtained using backward stepwise selection, included BMI, SARC-CalF, SPPB, and FMA motor impairment categories, adjusted for age, sex, and time since stroke. The model was statistically significant (χ²(9) = 86.144; p < 0.001), explained 59.0% of the variance in sarcopenia (Nagelkerke R² = 0.59), and showed acceptable fit (Hosmer–Lemeshow χ²(8) = 8.640; p = 0.374).

In the final multivariable model, higher BMI was independently associated with lower odds of sarcopenia (OR per 1 kg/m² increase = 0.70; 95% CI: 0.59–0.83), while positive SARC-CalF screening was associated with increased odds (OR = 6.37; 95% CI: 2.03–20.05). Higher SPPB scores were also associated with reduced odds of sarcopenia (OR per 1-point increase = 0.83; 95% CI: 0.69–0.99). Compared with severe motor impairment, participants with mild and moderately severe impairment had lower odds of sarcopenia, although the moderate category did not reach statistical significance. The model demonstrated good discriminative ability (AUC-ROC = 0.86; 95% CI: 0.81–0.91). Table 3 presents adjusted estimates from the logistic regression analysis.

**Table 3:** Multivariable logistic regression analysis of factors associated with sarcopenia.

Variable	Adjusted OR (95% CI)	p-value
BMI (per 1 kg/m² increase)	0.70 (0.59–0.83)	<0.001
SARC-CalF positive (≥11)	6.37 (2.03–20.05)	0.001
SPPB (per 1-point increase)	0.83 (0.69–0.99)	0.041
FMA category: moderately severe vs severe	0.07 (0.01–0.64)	0.018
FMA category: moderate vs severe	0.19 (0.03–1.17)	0.073
FMA category: mild vs severe	0.10 (0.01–0.91)	0.041

Adjusted for age, sex, and time since stroke. Reference category: severe motor impairment.

Model performance: AUC-ROC = 0.86 (95% CI: 0.81–0.91); Hosmer–Lemeshow p = 0.374.

## Discussion

This study shows that sarcopenia is common among individuals undergoing post-stroke rehabilitation and is consistently associated with lower BMI, poorer motor performance, reduced physical function, and lower levels of functional independence. Rather than representing an isolated condition, these findings support the interpretation of sarcopenia as a marker of a more impaired functional phenotype after stroke, reflecting the complex interaction between neurological injury, muscle dysfunction, nutritional status, and reduced physical activity.

The prevalence observed in this study is broadly consistent with previous systematic reviews and meta-analyses reporting rates between approximately 33% and 52% among stroke survivors [4,5]. However, the lower prevalence identified using DXA-confirmed criteria warrants careful interpretation. This may reflect the use of more stringent diagnostic thresholds, as well as differences in patient characteristics, including the inclusion of individuals undergoing active rehabilitation who may retain partial functional capacity. These findings highlight the influence of diagnostic approach and clinical context on sarcopenia prevalence and limit direct comparisons across studies.

Because the estimated prevalence of sarcopenia is strongly influenced by the diagnostic criteria adopted, it is important to consider the applicability of the consensus used in the present study. The EWGSOP2 consensus was originally developed for age-related sarcopenia in older adults and also recognizes secondary sarcopenia associated with disease, physical inactivity, or malnutrition [1]. Stroke is one of the clinical conditions in which secondary sarcopenia may develop. Currently, no internationally accepted diagnostic consensus specifically developed for post-stroke sarcopenia is available. Consequently, studies in this field have routinely adopted established diagnostic frameworks such as EWGSOP2, AWGS, IWGS, or the FNIH Sarcopenia Project. Recent literature has highlighted the limitations of applying these general diagnostic criteria to stroke populations and emphasized the need for stroke-specific diagnostic criteria [19,20]. Although the study included adults aged ≥18 years, the study population was predominantly older (mean age 60.6 ± 13.9 years), supporting the applicability of the EWGSOP2 criteria to the majority of participants.

In our sample, HGS and the 5STS identified substantially higher proportions of affected individuals than the DXA-based diagnostic criteria. Few studies have explored this discrepancy in individuals undergoing neurorehabilitation after stroke. This divergence supports the concept that impairments in muscle strength and physical performance may precede detectable reductions in muscle mass, representing an earlier and more clinically sensitive manifestation of sarcopenia [1]. In stroke populations, this dissociation is likely amplified by disease-specific mechanisms, including denervation, impaired motor unit recruitment, neuromuscular inefficiency, and central motor deficits [2,19]. Accordingly, the relationship between muscle mass and functional capacity appears moderate, as lean mass explains part, but not all, of the variability in gait performance and aerobic capacity [3]. Taken together, these findings support a function-centered interpretation of sarcopenia in neurological populations, in which functional impairment may represent a more clinically meaningful construct than muscle mass alone.

From a pathophysiological perspective, post-stroke sarcopenia appears to reflect the convergence of multiple mechanisms, including systemic inflammation, reduced physical activity, decreased nutritional intake, anabolic resistance, and impaired neuromuscular activation [7,20]. Within this framework, recovery after stroke depends not only on the extent of neurological injury but also on the integrity of peripheral systems responsible for movement execution. Muscle weakness and reduced physical capacity may limit training intensity and tolerance to repetitive practice, which are key drivers of neuroplasticity and functional recovery [10,21]. Therefore, sarcopenia may be more appropriately interpreted as a condition closely linked to functional limitation rather than a purely structural abnormality.

These findings have important implications for assessment and rehabilitation strategies. Reliance solely on muscle mass may underestimate clinically meaningful impairment, whereas functional measures capture a broader and more integrative spectrum of disability, encompassing both muscular and neural components. Consistent with previous studies, sarcopenia was associated with poorer motor performance and reduced functional independence [8,22]. HGS is widely recognized as a reliable indicator [23] of overall muscle strength and is recommended by the EWGSOP2 as the primary measure for identifying probable sarcopenia [1]. However, reduced HGS was substantially more frequent than DXA-confirmed low muscle mass in the present study, suggesting that muscle weakness may be present even in individuals who do not yet meet imaging-based criteria for sarcopenia.

The strong association between lower BMI and sarcopenia observed in this study further highlights the potential role of nutritional and metabolic factors. Lower BMI may reflect reduced nutritional reserves, reduced muscle mass, a catabolic state, or other metabolic alterations, which can be exacerbated by dysphagia, reduced dietary intake, systemic inflammation, and physical inactivity following stroke [24,25]. Although BMI is an easily obtainable clinical measure, it should not be interpreted as a direct measure of muscle mass or as a comprehensive assessment of nutritional status. In addition, excess adiposity may coexist with reduced muscle mass (sarcopenic obesity), reinforcing that BMI alone is insufficient to characterize body composition and highlighting the importance of direct body composition assessment for the accurate identification of low muscle mass [26,27]. Because validated nutritional assessment tools, such as the Mini Nutritional Assessment (MNA) or the Global Leadership Initiative on Malnutrition (GLIM) criteria, as well as dietary intake and inflammatory biomarkers, were not collected, the mechanisms underlying the observed association between BMI and sarcopenia cannot be fully elucidated.

Accumulating evidence supports the central role of energy and protein intake in modulating muscle mass and functional recovery. Positive energy balance during hospitalization has been associated with increases in skeletal muscle mass [25], while higher energy intake combined with greater rehabilitation intensity has been associated with a lower risk of hospital-acquired sarcopenia and improved functional outcomes [24,28]. Additional studies indicate that poor nutritional status, lower BMI, and inadequate weight maintenance are associated with reduced functional gains [29,30], whereas greater rehabilitation intensity may attenuate BMI decline during recovery [31]. Taken together, these findings suggest that lower BMI may represent a clinical marker of metabolic vulnerability associated with sarcopenia and poorer functional outcomes following stroke.

The observed association between sarcopenia and poorer performance on both the FMA and the SPPB reinforces its relevance to functional recovery. These instruments capture complementary domains of function, including motor impairment assessed by the FMA and balance, mobility, and lower extremity strength assessed by the SPPB. The concurrent impairment across these domains suggests that sarcopenia reflects a global reduction in functional capacity rather than isolated muscle loss. Previous studies have similarly demonstrated that reduced muscle mass is associated with poorer gait performance, slower walking speed, and reduced independence after stroke [32,33].

Importantly, the influence of sarcopenia extends beyond baseline status and appears to affect recovery trajectories. Patients with sarcopenia tend to present with worse functional status, achieve smaller functional gains, and have a lower likelihood of discharge to home [8,34,35]. Conversely, increases in muscle mass during rehabilitation and reversal of sarcopenia have been associated with improved functional outcomes and discharge status [22,36]. These findings indicate that sarcopenia is not only a prognostic marker but also a clinically actionable target. However, because of the cross-sectional design, causal relationships between sarcopenia and functional impairment cannot be established.

These results support the integration of systematic sarcopenia screening into routine clinical practice. Functional measures may serve as early indicators of risk, particularly in patients with low BMI or reduced physical performance.

Interventional studies further reinforce the modifiable nature of sarcopenia and rehabilitation programs should incorporate multimodal strategies combining resistance training, nutritional optimization, and task-specific functional training to address both neurological and musculoskeletal components of recovery [11,37,38]. A multidisciplinary approach is essential to manage the multifactorial nature of post-stroke sarcopenia.

### Limitations

The cross-sectional design precludes causal inference, and the single-centre setting may limit generalisability. In addition, potentially relevant factors, including inflammatory markers, hormonal status, detailed dietary intake, and personal characteristics such as educational level and socioeconomic status were not included in the analysis. Future longitudinal studies are needed to clarify the temporal relationship between sarcopenia and functional recovery and to evaluate the effectiveness of targeted interventions.

A proportion of eligible patients were excluded because DXA was not requested, despite physicians having been instructed to request DXA for all eligible patients with stroke. This reflected incomplete implementation of the assessment protocol rather than selective referral based on suspected sarcopenia. Furthermore, no significant differences in age or mRS scores were observed between included and excluded participants. Nevertheless, potential selection bias cannot be completely excluded, and the findings should be interpreted with this limitation in mind.

Because participants with severe cognitive and motor impairments were not assessed using the FMA and FIM according to the institutional assessment protocol, analyses involving these variables excluded this subgroup. Consequently, the findings related to these measures should be interpreted with caution, particularly regarding their applicability to individuals with severe cognitive and motor impairments.

## Conclusion

DXA-confirmed post-stroke sarcopenia affected approximately one in four individuals undergoing rehabilitation and was independently associated with lower BMI, poorer physical performance, and greater motor impairment. These findings indicate that sarcopenia is closely linked to functional impairment after stroke and reinforce the importance of integrating muscle health and functional assessment during rehabilitation. Early identification of individuals at risk may support more individualized rehabilitation strategies and improve clinical decision-making.
