# Supplementary material for: Prevalence and factors associated with sarcopenia in individuals with stroke undergoing rehabilitation: A cross-sectional study
Source: PLoS One. 2026 Oct 26;21(10):e0361113. doi: 10.1371/journal.pone.0361113 (PMC13704826; doi:10.1371/journal.pone.0361113)
Supplement: Supplementary file 1 [file pone.0361113.s001.docx]

Reviewer 1

The manuscript addresses an important clinical issue by investigating the association between sarcopenia and functional outcomes among patients undergoing stroke rehabilitation. The study is generally well designed and contributes valuable information to the growing body of literature on post-stroke sarcopenia. However, several methodological and reporting issues should be addressed to improve the transparency, validity, and interpretability of the findings.
Minor Comments
1. Insufficient Description of DXA Measurement Quality Control
Appendicular skeletal muscle index (ASMI), the primary parameter used to diagnose sarcopenia, was assessed using dual-energy X-ray absorptiometry (DXA). However, the manuscript does not provide information regarding quality assurance procedures, calibration protocols, or operator training. Since DXA-derived measurements constitute a key study outcome, additional details regarding measurement reliability are warranted.
Recommendation: The authors should consider adding a statement such as:
"DXA scans were performed according to the manufacturer's standardized protocol. Daily calibration procedures were conducted before participant assessment, and scans were analysed by trained personnel blinded to clinical outcomes."
This would provide reassurance that measurement bias was minimized and that the DXA assessments were performed under standardized conditions.
2. Extent of Missing Data Is Not Reported
The Methods section states that a complete-case analysis approach was used to handle missing data; however, the overall proportion of missing observations is not reported. Without this information, readers cannot adequately assess the potential impact of missing data on the study findings.
Recommendation: The authors should report the percentage of missing data either in the Methods or Results section. For example:
"The proportion of missing data for all variables was less than 5%."
or
"No variable had more than 3% missing observations."
Providing this information would improve transparency and facilitate the assessment of potential bias.
3. Incomplete Reporting of Missing Data in the Results Section
The Results section indicates different sample sizes across variables (e.g., SARC-CalF, n = 203; HGS, n = 207; FMA, n = 196; FIM, n = 183), suggesting that some observations were missing. However, the manuscript does not explain the reasons for missing data, the percentage of missing observations, or the number of participants ultimately included in the multivariable logistic regression analyses.
Recommendation: The authors should clarify the final analytical sample size by including a statement such as:
"The final multivariable model included 183 participants with complete data."
Additional details regarding the pattern and handling of missing data would further strengthen the reporting quality.
4. Interpretation of Findings Should Reflect the Cross-Sectional Study Design
Several statements in the Discussion imply causal relationships, including assertions that lower BMI contributes to muscle loss and impaired recovery, that sarcopenia affects recovery trajectories, and that muscle weakness may limit rehabilitation intensity. While these interpretations are biologically plausible, the cross-sectional design precludes establishing temporal or causal relationships.
Recommendation: The discussion should be revised to emphasize associations rather than causation. The authors may consider adding the following statement:
"Because of the cross-sectional design, causal relationships between sarcopenia and functional impairment cannot be established."
This clarification would ensure that the interpretation of the findings remains consistent with the study design.
5. Potential Selection Bias Should Be Acknowledged
The limitations section would benefit from a discussion of potential selection bias. A considerable number of potentially eligible patients appear to have been excluded because DXA assessment was not requested or not completed. Consequently, the final study sample may not fully represent the broader population of patients undergoing stroke rehabilitation.
Recommendation: The authors may consider adding the following statement:
"A substantial proportion of eligible participants were excluded because DXA assessment was not requested or completed, which may have introduced selection bias."
Acknowledging this issue would provide a more balanced interpretation of the study findings and improve the transparency of the manuscript.
Overall Assessment
The study addresses a clinically relevant topic and provides valuable evidence regarding the association between sarcopenia and functional outcomes in patients undergoing stroke rehabilitation. Addressing the issues outlined above would substantially strengthen the methodological transparency and scientific rigor of the manuscript and enhance readers' confidence in the reported findings.

Reviewer 2

Thank you for the opportunity to review this manuscript. The study addresses an important and clinically relevant topic and benefits from the use of DXA-based sarcopenia assessment in a relatively large cohort of individuals undergoing stroke rehabilitation. The manuscript is generally well organized and the analyses are clearly presented. However, several issues should be addressed before publication.

A substantial proportion of eligible participants were excluded because DXA examination was not requested (102 of 311 eligible patients). This may have introduced selection bias. The authors should clarify how DXA referral decisions were made and discuss the potential impact of this exclusion on the study findings.
The study included adults aged ≥18 years. Since EWGSOP2 criteria were primarily developed and validated in older populations, the applicability of these cut-offs to younger stroke survivors should be discussed more thoroughly.
Nutritional and inflammatory variables were not evaluated. Given that lower BMI emerged as an independent factor associated with sarcopenia, the absence of nutritional assessments (e.g., MNA, GLIM criteria, dietary intake) and inflammatory markers should be emphasized when interpreting the results.
Although the study identifies factors associated with sarcopenia, its cross-sectional design precludes causal inference. This limitation should be highlighted more clearly in the discussion.
The discussion may be strengthened by incorporating additional literature regarding muscle mass assessment and sarcopenic obesity:
Yilmaz O, Bahat G. Suggestions for assessment of muscle mass in primary care setting. Aging Male. 2017;20(3):168-169. doi:10.1080/13685538.2017.1311856.
Yilmaz O, Idiz C, Kilic C, Yildiz B, Bahat G. Screening risk of sarcopenic obesity using SARC-F and handgrip strength in outpatients with diabetes. Metab Syndr Relat Disord. 2026;24(1):17-25. doi:10.1177/15578518251386225.
