# Supplementary material for: Prevalence and factors associated with sarcopenia in individuals with stroke undergoing rehabilitation: A cross-sectional study
Source: PLoS One. 2026 Oct 26;21(10):e0361113. doi: 10.1371/journal.pone.0361113 (PMC13704826; doi:10.1371/journal.pone.0361113)
Supplement: Supplementary file 2 [file pone.0361113.s003.docx]

# ***RESPONSE TO REVIEWERS***

***Manuscript ID:*** *PONE-D-26-22047*

***Title:*** *Prevalence and factors associated with sarcopenia in individuals with stroke undergoing rehabilitation: a cross-sectional study*

## ***Dear Academic Editor,***

*We sincerely thank you and the reviewers for the careful evaluation of our manuscript and for the thoughtful and constructive comments. We greatly appreciate the time and expertise devoted to reviewing our work.*

*We have carefully considered every comment and revised the manuscript accordingly. The reviewers' suggestions have substantially improved the methodological transparency, scientific rigor, and clarity of the manuscript.*

*Detailed point-by-point responses are provided below. All revisions are highlighted using Track Changes in the revised manuscript.*

# ***Reviewer 1***

*We sincerely thank the reviewer for the thorough assessment of our manuscript and for the valuable suggestions. The comments were highly constructive and helped us improve both the methodological reporting and the overall clarity of the study. Our detailed responses are provided below.*

# ***Comment 1***

*Appendicular skeletal muscle index (ASMI), the primary parameter used to diagnose sarcopenia, was assessed using dual-energy X-ray absorptiometry (DXA). However, the manuscript does not provide information regarding quality assurance procedures, calibration protocols, or operator training. Since DXA-derived measurements constitute a key study outcome, additional details regarding measurement reliability are warranted.*

### ***Response***

*We thank the reviewer for this valuable suggestion.*

*To improve methodological transparency, we expanded the* ***Methods*** *section by providing a more detailed description of the DXA acquisition procedures, operator training, quality control, and calibration protocol.*

*Specifically, we clarified that DXA examinations were performed by a trained radiology technician under the supervision of a board-certified radiologist. We also added information describing the quality assurance procedures adopted throughout the study, including daily quality control and weekly calibration using the manufacturer's phantom according to the manufacturer's recommendations. Furthermore, we clarified that all examinations followed a standardized patient positioning protocol and that both professionals were blinded to the study objectives and participants' clinical and functional assessments.*

*These additions provide a more complete description of the procedures implemented to ensure the reliability and reproducibility of DXA measurements.*

***Location of revision:*** ***Methods, page 5, lines 97–105.***

# ***Comment 2***

*The Methods section states that a complete-case analysis approach was used to handle missing data; however, the overall proportion of missing observations is not reported. Without this information, readers cannot adequately assess the potential impact of missing data on the study findings.*

### ***Response***

*We appreciate this important observation.*

*To improve transparency, we expanded the description of missing data in the* ***Statistical Analysis*** *section.*

*Specifically, we clarified that missing data were handled using a complete-case analysis approach, that no imputation procedures were performed, and that missing data due to incomplete assessments were infrequent (<3% for all variables). We also distinguished planned non-assessments performed according to the institutional assessment protocol (i.e., FIM and Fugl-Meyer Assessment in participants with severe cognitive and motor impairments) from missing data resulting from incomplete assessments.*

*These revisions provide readers with a clearer understanding of the completeness of the dataset and the analytical approach adopted.*

***Location of revision:*** ***Methods (Statistical analysis), page 8, lines 171–177.***

# ***Comment 3***

*The Results section indicates different sample sizes across variables (e.g., SARC-CalF, n = 203; HGS, n = 207; FMA, n = 196; FIM, n = 183), suggesting that some observations were missing. However, the manuscript does not explain the reasons for missing data, the percentage of missing observations, or the number of participants ultimately included in the multivariable logistic regression analyses.*

### ***Response***

*We thank the reviewer for this important comment.*

*To improve transparency regarding data availability and sample sizes across analyses, we revised the manuscript in several sections.*

*First, in the* ***Methods****, we clarified that the Fugl-Meyer Assessment (FMA) and the Functional Independence Measure (FIM) were not administered to participants with severe cognitive and motor impairments when reliable standardized assessment was not feasible, in accordance with the institutional assessment protocol.*

*Second, we expanded the* ***Statistical Analysis*** *section to clarify that multivariable logistic regression analyses were conducted using a complete-case approach, including only participants with complete data for all variables entered into the final model.*

*Third, we clarified that missing data due to incomplete assessments were infrequent (<3% for all variables) and explicitly distinguished these observations from planned non-assessments performed according to the institutional assessment protocol.*

*Finally, we revised* ***Table 1*** *and* ***Table 2*** *to report the number of participants available for variables with incomplete assessments (e.g., FMA and FIM), thereby allowing readers to readily identify differences in sample size across analyses. We also addressed this issue in the* ***Limitations*** *section by clarifying that analyses involving the FMA and FIM excluded participants with severe cognitive and motor impairments and that findings related to these measures should therefore be interpreted with appropriate caution.*

*Collectively, these revisions improve the transparency of data reporting and facilitate interpretation of both the descriptive and multivariable analyses.*

***Locations of revision:***

- ***Methods (Clinical and functional variables), page 6, lines 135–137.***
- ***Methods (Statistical analysis), page 7, lines 158–180 and page 8, lines 171–177.***
- ***Tables 1 and 2, pages 11–13.***
- ***Limitations, page 29, lines 377–381***

## ***Comment 4***

*The cross-sectional design limits the ability to establish temporal or causal relationships between sarcopenia and the associated factors identified in the multivariable analyses. This limitation should be more explicitly acknowledged in both the Discussion and Limitations sections.*

### ***Response***

*We thank the reviewer for this important observation.*

*We agree that the cross-sectional design precludes establishing temporal or causal relationships between sarcopenia and the associated variables. To address this comment, we revised both the* ***Discussion*** *and* ***Limitations*** *sections to explicitly acknowledge this limitation and to ensure that the interpretation of the findings remains consistent with the observational nature of the study.*

*Specifically, we clarified that the identified associations should not be interpreted as evidence of causality or directionality and emphasized that longitudinal studies are needed to determine the temporal relationships between sarcopenia and the clinical and functional factors identified in the present study.*

*These revisions strengthen the interpretation of our findings and provide a more balanced discussion of the study's methodological limitations.*

***Locations of revision:***

- ***Discussion, page 18, lines 353–354.***
- ***Limitations, page 19, lines 364–365.***

# ***Comment 5***

*Approximately one-third of eligible participants were excluded because DXA was not requested. This raises concerns regarding possible selection bias. Please clarify whether these exclusions may have influenced the study findings and discuss this issue as a study limitation.*

### ***Response***

*We sincerely thank the reviewer for this valuable comment.*

*According to the study protocol, all attending physicians were instructed to request DXA for every eligible participant with stroke upon admission to the rehabilitation program, regardless of clinical suspicion of sarcopenia. However, DXA was not requested for 85 participants because the assessment protocol was not consistently implemented during routine clinical practice.*

*To further evaluate the potential impact of these exclusions, we compared included and excluded participants and found no significant differences in age or modified Rankin Scale (mRS) scores. We have now reported these findings in the* ***Results*** *section.*

*Nevertheless, because unmeasured differences between included and excluded participants cannot be entirely excluded, we have explicitly acknowledged the possibility of residual selection bias in the* ***Limitations*** *section and advised that the findings should be interpreted in light of this consideration.*

*These revisions improve the transparency of participant selection and provide readers with a more balanced interpretation of the study findings.*

***Locations of revision:***

- ***Methods, page 5, lines 97–100.***
- ***Results, page 9, lines 184–189.***
- ***Limitations, page 19, lines 371–377.***

# ***Reviewer 2***

*We sincerely thank the reviewer for the careful evaluation of our manuscript and for the thoughtful comments. We greatly appreciate the constructive suggestions, which have helped improve the scientific interpretation and reporting of our findings. Our detailed responses are provided below.*

# ***Comment 1***

*Approximately 30% of eligible patients were excluded because DXA was not requested. Although the authors report no differences in age or mRS between included and excluded participants, additional clarification is needed regarding the possibility of selection bias.*

### ***Response***

*We sincerely thank the reviewer for this valuable comment.*

*According to the study protocol, all attending physicians were instructed to request DXA for every eligible participant with stroke upon admission to the rehabilitation program, regardless of clinical suspicion of sarcopenia. However, DXA was not requested for 85 participants because the assessment protocol was not consistently implemented during routine clinical practice.*

*To further evaluate the potential impact of these exclusions, we compared included and excluded participants and found no significant differences in age or modified Rankin Scale (mRS) scores. We have now reported these findings in the* ***Results*** *section.*

*Nevertheless, because unmeasured differences between included and excluded participants cannot be entirely excluded, we have explicitly acknowledged the possibility of residual selection bias in the* ***Limitations*** *section and advised that the findings should be interpreted in light of this consideration.*

*These revisions improve the transparency of participant selection and provide readers with a more balanced interpretation of the study findings.*

***Locations of revision:***

- ***Methods, page 5, lines 97–100.***
- ***Results, page 9, lines 184–189.***
- ***Limitations, page 19, lines 371–377.***

# ***Comment 2***

*The applicability of the EWGSOP2 criteria to individuals with stroke should be discussed in greater depth.*

### ***Response***

*We thank the reviewer for this valuable suggestion.*

*To strengthen the Discussion, we expanded the section addressing the applicability of current diagnostic criteria for sarcopenia in stroke populations.*

*Specifically, we clarified that the* ***EWGSOP2*** *consensus was originally developed for age-related sarcopenia while also recognizing secondary sarcopenia associated with chronic diseases, including stroke. We further emphasized that no internationally accepted diagnostic criteria specifically developed for post-stroke sarcopenia are currently available. Consequently, previous studies have adopted established diagnostic frameworks such as the* ***EWGSOP2****,* ***AWGS****,* ***IWGS****, and* ***FNIH*** *criteria.*

*We also incorporated recent literature highlighting the limitations of applying general sarcopenia definitions to stroke populations and the need for stroke-specific diagnostic criteria. Finally, we clarified that our study population was predominantly composed of older adults (mean age 63.0 ± 13.9 years), which supports the applicability of the EWGSOP2 criteria within the context of the present study.*

*These additions provide broader clinical context for interpreting our findings while acknowledging the current limitations of available diagnostic frameworks.*

***Location of revision:***

- ***Discussion, page 15, lines 273–286.***

# ***Comment 3***

*Nutritional and inflammatory variables were not evaluated. Given that lower BMI emerged as an independent factor associated with sarcopenia, the absence of nutritional assessments (e.g., MNA, GLIM criteria, dietary intake) and inflammatory markers should be emphasized when interpreting the results.*

### ***Response***

*We sincerely thank the reviewer for this important observation.*

*We agree that nutritional status and systemic inflammation are important contributors to sarcopenia and should be considered when interpreting the observed association between lower BMI and sarcopenia.*

*To address this comment, we expanded the* ***Discussion*** *in three ways. First, we clarified that BMI should not be interpreted as a direct measure of muscle mass or nutritional status. Second, we incorporated additional discussion regarding sarcopenic obesity, emphasizing that excess adiposity may coexist with reduced muscle mass and that BMI alone is insufficient to characterize body composition. Finally, we explicitly acknowledged that validated nutritional assessment tools (e.g., Mini Nutritional Assessment [MNA] and Global Leadership Initiative on Malnutrition [GLIM] criteria), dietary intake, and inflammatory biomarkers were not collected. Consequently, the mechanisms underlying the observed association between lower BMI and sarcopenia cannot be fully elucidated.*

*These revisions provide a more balanced interpretation of the association between BMI and sarcopenia while clearly acknowledging the limitations of the available data.*

***Location of revision:***

- ***Discussion, page 17, lines 317–340.***

## ***Comment 4***

*Because of the cross-sectional design, causal relationships between sarcopenia and the associated variables cannot be established. This limitation should be more explicitly acknowledged.*

### ***Response***

*We thank the reviewer for this important comment.*

*We agree that the cross-sectional design precludes establishing temporal or causal relationships between sarcopenia and the associated variables identified in our analyses.*

*To address this comment, we revised both the* ***Discussion*** *and* ***Limitations*** *sections to explicitly acknowledge this methodological limitation. We clarified that the observed associations should not be interpreted as evidence of causality or temporal sequence and emphasized that prospective longitudinal studies are needed to determine the directionality of these relationships.*

*These revisions ensure that the interpretation of our findings remains fully consistent with the observational design of the present study.*

***Locations of revision:***

- ***Discussion, page 18, lines 353–354.***
- ***Limitations, page 19, lines 364–365.***

# ***Comment 5***

*The discussion may be strengthened by incorporating additional literature regarding muscle mass assessment and sarcopenic obesity.*

### ***Response***

*We sincerely thank the reviewer for this valuable suggestion.*

*We incorporated the recommended article by* ***Yilmaz O, Idiz C, Kilic C, Yildiz B, and Bahat G.*** *(Screening risk of sarcopenic obesity using SARC-F and handgrip strength in outpatients with diabetes. Metabolic Syndrome and Related Disorders. 2025) into the* ***Discussion****.*

*Specifically, we expanded the interpretation of the association between BMI and sarcopenia by acknowledging that excess adiposity may coexist with reduced muscle mass (sarcopenic obesity), reinforcing that BMI alone is insufficient to characterize body composition. We also emphasized the importance of direct body composition assessment for accurately identifying low muscle mass, while acknowledging that nutritional assessment and inflammatory biomarkers were not available in the present study.*

*These additions provide additional context for interpreting the observed association between BMI and sarcopenia and strengthen the discussion of body composition assessment.*

***Location of revision:***

- ***Discussion, page 17, lines 317–330.***

# ***Closing Statement***

*Once again, we sincerely thank the Academic Editor and both reviewers for their thoughtful comments and constructive suggestions. Their feedback has substantially improved the methodological transparency, scientific rigor, and clarity of our manuscript.*

*We have carefully addressed every comment and believe that the revised manuscript provides a more robust and transparent presentation of our study. We hope that the revised version will be suitable for publication in* ***PLOS ONE****.*

*Sincerely,*

***Fernando Ida***
 *(on behalf of all authors)*
