# Supplementary material for: Prevalence and factors associated with sarcopenia in individuals with stroke undergoing rehabilitation: A cross-sectional study
Source: PLoS One. 2026 Oct 26;21(10):e0361113. doi: 10.1371/journal.pone.0361113 (PMC13704826; doi:10.1371/journal.pone.0361113)
Supplement: Peer review history

# Peer review: Decision Letter 0

**DOI:** 10.1371/journal.pone.0361113.r001

**Authors:** Myadagmaa Jaalkhorol

29 Jun 2026

Dear Dr. Ida,

Thank you for submitting your manuscript to PLOS ONE. After careful consideration, we feel that it has merit but does not fully meet PLOS ONE’s publication criteria as it currently stands. Therefore, we invite you to submit a revised version of the manuscript that addresses the points raised during the review process.

Please submit your revised manuscript by Aug 28 2026 11:59PM. If you will need more time than this to complete your revisions, please reply to this message or contact the journal office at plosone@plos.org. When you're ready to submit your revision, log on to https://www.editorialmanager.com/pone/ and select the 'Submissions Needing Revision' folder to locate your manuscript file.

- A letter that responds to each point raised by the academic editor and reviewer(s). You should upload this letter as a separate file labeled 'Response to Reviewers'.
- A marked-up copy of your manuscript that highlights changes made to the original version. You should upload this as a separate file labeled 'Revised Manuscript with Track Changes'.
- An unmarked version of your revised paper without tracked changes. You should upload this as a separate file labeled 'Manuscript'.

If you would like to make changes to your financial disclosure, please include your updated statement in your cover letter. Guidelines for resubmitting your figure files are available below the reviewer comments at the end of this letter.

If applicable, we recommend that you deposit your laboratory protocols in protocols.io to enhance the reproducibility of your results. Protocols.io assigns your protocol its own identifier (DOI) so that it can be cited independently in the future. For instructions see: https://journals.plos.org/plosone/s/submission-guidelines#loc-laboratory-protocols. Additionally, PLOS ONE offers an option for publishing peer-reviewed Lab Protocol articles, which describe protocols hosted on protocols.io. Read more information on sharing protocols at https://plos.org/protocols?utm_medium=editorial-email&utm_source=authorletters&utm_campaign=protocols.

As the corresponding author, your ORCID iD is verified in the submission system and will appear in the published article. PLOS supports the use of ORCID, and we encourage all coauthors to register for an ORCID iD and use it as well. Please encourage your coauthors to verify their ORCID iD within the submission system before final acceptance, as unverified ORCID iDs will not appear in the published article. *Only*  the individual author can complete the verification step; PLOS staff *cannot*  verify ORCID iDs on behalf of authors.

We look forward to receiving your revised manuscript.

Kind regards,

Myadagmaa Jaalkhrol

Academic Editor

PLOS One

Journal Requirements:

When submitting your revision, we need you to address these additional requirements.

1. Please ensure that your manuscript meets PLOS ONE's style requirements, including those for file naming. The PLOS ONE style templates can be found at

https://journals.plos.org/plosone/s/file?id=wjVg/PLOSOne_formatting_sample_main_body.pdf and https://journals.plos.org/plosone/s/file?id=ba62/PLOSOne_formatting_sample_title_authors_affiliations.pdf

2. In the online submission form, you indicated that all relevant data are within the manuscript. De-identified participant data may be made available upon reasonable request to the corresponding author.

All PLOS journals now require all data underlying the findings described in their manuscript to be freely available to other researchers, either 1. In a public repository, 2. Within the manuscript itself, or 3. Uploaded as supplementary information.

This policy applies to all data except where public deposition would breach compliance with the protocol approved by your research ethics board. If your data cannot be made publicly available for ethical or legal reasons (e.g., public availability would compromise patient privacy), please explain your reasons on resubmission and your exemption request will be escalated for approval.

3. Please ensure that you refer to Figure 1 in your text as, if accepted, production will need this reference to link the reader to the figure.

4. We note you have included a table to which you do not refer in the text of your manuscript. Please ensure that you refer to Table 1, 2 in your text; if accepted, production will need this reference to link the reader to the Table.

Additional Editor Comments:

Dear Dr, Fernando Shizuo Ida

You can edit and submit according to the comments given by the reviewers.

Academic Editor

Myadagmaa Jaalkhorol

[Note: HTML markup is below. Please do not edit.]

Reviewers' comments:

Reviewer's Responses to Questions

**Comments to the Author**

1. Is the manuscript technically sound, and do the data support the conclusions?

Reviewer #1: Yes

Reviewer #2: Yes

**********

2. Has the statistical analysis been performed appropriately and rigorously?

Reviewer #1: N/A

Reviewer #2: Yes

**********

3. Have the authors made all data underlying the findings in their manuscript fully available?

The PLOS Data policy

Reviewer #1: Yes

Reviewer #2: Yes

**********

4. Is the manuscript presented in an intelligible fashion and written in standard English?

Reviewer #1: Yes

Reviewer #2: Yes

**********

Reviewer #1: Reviewer Comments

The manuscript addresses an important clinical issue by investigating the association between sarcopenia and functional outcomes among patients undergoing stroke rehabilitation. The study is generally well designed and contributes valuable information to the growing body of literature on post-stroke sarcopenia. However, several methodological and reporting issues should be addressed to improve the transparency, validity, and interpretability of the findings.

Minor Comments

1. Insufficient Description of DXA Measurement Quality Control

Appendicular skeletal muscle index (ASMI), the primary parameter used to diagnose sarcopenia, was assessed using dual-energy X-ray absorptiometry (DXA). However, the manuscript does not provide information regarding quality assurance procedures, calibration protocols, or operator training. Since DXA-derived measurements constitute a key study outcome, additional details regarding measurement reliability are warranted.

Recommendation: The authors should consider adding a statement such as:

"DXA scans were performed according to the manufacturer's standardized protocol. Daily calibration procedures were conducted before participant assessment, and scans were analysed by trained personnel blinded to clinical outcomes."

This would provide reassurance that measurement bias was minimized and that the DXA assessments were performed under standardized conditions.

2. Extent of Missing Data Is Not Reported

The Methods section states that a complete-case analysis approach was used to handle missing data; however, the overall proportion of missing observations is not reported. Without this information, readers cannot adequately assess the potential impact of missing data on the study findings.

Recommendation: The authors should report the percentage of missing data either in the Methods or Results section. For example:

"The proportion of missing data for all variables was less than 5%."

or

"No variable had more than 3% missing observations."

Providing this information would improve transparency and facilitate the assessment of potential bias.

3. Incomplete Reporting of Missing Data in the Results Section

The Results section indicates different sample sizes across variables (e.g., SARC-CalF, n = 203; HGS, n = 207; FMA, n = 196; FIM, n = 183), suggesting that some observations were missing. However, the manuscript does not explain the reasons for missing data, the percentage of missing observations, or the number of participants ultimately included in the multivariable logistic regression analyses.

Recommendation: The authors should clarify the final analytical sample size by including a statement such as:

"The final multivariable model included 183 participants with complete data."

Additional details regarding the pattern and handling of missing data would further strengthen the reporting quality.

4. Interpretation of Findings Should Reflect the Cross-Sectional Study Design

Several statements in the Discussion imply causal relationships, including assertions that lower BMI contributes to muscle loss and impaired recovery, that sarcopenia affects recovery trajectories, and that muscle weakness may limit rehabilitation intensity. While these interpretations are biologically plausible, the cross-sectional design precludes establishing temporal or causal relationships.

Recommendation: The discussion should be revised to emphasize associations rather than causation. The authors may consider adding the following statement:

"Because of the cross-sectional design, causal relationships between sarcopenia and functional impairment cannot be established."

This clarification would ensure that the interpretation of the findings remains consistent with the study design.

5. Potential Selection Bias Should Be Acknowledged

The limitations section would benefit from a discussion of potential selection bias. A considerable number of potentially eligible patients appear to have been excluded because DXA assessment was not requested or not completed. Consequently, the final study sample may not fully represent the broader population of patients undergoing stroke rehabilitation.

Recommendation: The authors may consider adding the following statement:

"A substantial proportion of eligible participants were excluded because DXA assessment was not requested or completed, which may have introduced selection bias."

Acknowledging this issue would provide a more balanced interpretation of the study findings and improve the transparency of the manuscript.

Overall Assessment

The study addresses a clinically relevant topic and provides valuable evidence regarding the association between sarcopenia and functional outcomes in patients undergoing stroke rehabilitation. Addressing the issues outlined above would substantially strengthen the methodological transparency and scientific rigor of the manuscript and enhance readers' confidence in the reported findings.

Reviewer #2: Thank you for the opportunity to review this manuscript. The study addresses an important and clinically relevant topic and benefits from the use of DXA-based sarcopenia assessment in a relatively large cohort of individuals undergoing stroke rehabilitation. The manuscript is generally well organized and the analyses are clearly presented. However, several issues should be addressed before publication.

A substantial proportion of eligible participants were excluded because DXA examination was not requested (102 of 311 eligible patients). This may have introduced selection bias. The authors should clarify how DXA referral decisions were made and discuss the potential impact of this exclusion on the study findings.

The study included adults aged ≥18 years. Since EWGSOP2 criteria were primarily developed and validated in older populations, the applicability of these cut-offs to younger stroke survivors should be discussed more thoroughly.

Nutritional and inflammatory variables were not evaluated. Given that lower BMI emerged as an independent factor associated with sarcopenia, the absence of nutritional assessments (e.g., MNA, GLIM criteria, dietary intake) and inflammatory markers should be emphasized when interpreting the results.

Although the study identifies factors associated with sarcopenia, its cross-sectional design precludes causal inference. This limitation should be highlighted more clearly in the discussion.

The discussion may be strengthened by incorporating additional literature regarding muscle mass assessment and sarcopenic obesity:

Yilmaz O, Bahat G. Suggestions for assessment of muscle mass in primary care setting. Aging Male. 2017;20(3):168-169. doi:10.1080/13685538.2017.1311856.

Yilmaz O, Idiz C, Kilic C, Yildiz B, Bahat G. Screening risk of sarcopenic obesity using SARC-F and handgrip strength in outpatients with diabetes. Metab Syndr Relat Disord. 2026;24(1):17-25. doi:10.1177/15578518251386225.

**********

what does this mean?). If published, this will include your full peer review and any attached files.

If you choose “no”, your identity will remain anonymous but your review may still be made public.

**Do you want your identity to be public for this peer review?**  For information about this choice, including consent withdrawal, please see our Privacy Policy

Reviewer #1: **Yes:**  Odonchimeg Purev

Reviewer #2: **Yes:**  Ozlem Yılmaz

**********

[NOTE: If reviewer comments were submitted as an attachment file, they will be attached to this email and accessible via the submission site. Please log into your account, locate the manuscript record, and check for the action link "View Attachments". If this link does not appear, there are no attachment files.]

To ensure your figures meet our technical requirements, please review our figure guidelines: https://journals.plos.org/plosone/s/figures

You may also use PLOS’s free figure tool, NAAS, to help you prepare publication quality figures: https://journals.plos.org/plosone/s/figures#loc-tools-for-figure-preparation.

NAAS will assess whether your figures meet our technical requirements by comparing each figure against our figure specifications.

Attachment Submitted filename: Reviewers COMMENTS.docx

# Author comment: Author response to Decision Letter 1

**DOI:** 10.1371/journal.pone.0361113.r002

17 Sep 2026

Dear Academic Editor,

We sincerely thank you and the reviewers for the careful evaluation of our manuscript and for the constructive comments and suggestions. We have carefully considered all comments and have revised the manuscript accordingly.

A detailed point-by-point response to each comment is provided in the attached Response to Reviewers document. We have also uploaded a Revised Manuscript with Track Changes, showing all modifications made in response to the reviewers' comments, together with a clean version of the revised manuscript.

We believe that the revisions have strengthened the manuscript by improving its methodological transparency, clarity, and interpretation of the findings. We appreciate the opportunity to revise our work and hope that the revised version will be suitable for publication in PLOS ONE.

Sincerely,

Fernando Ida, on behalf of all authors

Attachment Submitted filename: RESPONSE TO REVIEWERS.docx

# Peer review: Decision Letter 1

**DOI:** 10.1371/journal.pone.0361113.r003

**Authors:** Myadagmaa Jaalkhorol

7 Oct 2026

Prevalence and factors associated with sarcopenia in individuals with stroke undergoing rehabilitation: a cross-sectional study

PONE-D-26-22047R1

Dear Dr.Fernando Shizuo Ida,

We’re pleased to inform you that your manuscript has been judged scientifically suitable for publication and will be formally accepted for publication once it meets all outstanding technical requirements.

Within one week, you’ll receive an e-mail detailing the required amendments. When these have been addressed, you’ll receive a formal acceptance letter and your manuscript will be scheduled for publication.

An invoice will be generated when your article is formally accepted. Please note, if your institution has a publishing partnership with PLOS and your article meets the relevant criteria, all or part of your publication costs will be covered. Please make sure your user information is up-to-date by logging into Editorial Manager at Editorial Manager® and clicking the ‘Update My Information' link at the top of the page. For questions related to billing, please contact billing support.

If your institution or institutions have a press office, please notify them about your upcoming paper to help maximize its impact. If they’ll be preparing press materials, please inform our press team as soon as possible -- no later than 48 hours after receiving the formal acceptance. Your manuscript will remain under strict press embargo until 2 pm Eastern Time on the date of publication. For more information, please contact onepress@plos.org.

Kind regards,

Myadagmaa Jaalkhorol, MD,PhD

Academic Editor

PLOS One

Reviewers' comments:

Reviewer's Responses to Questions

**Comments to the Author**

Reviewer #1: All comments have been addressed

Reviewer #2: All comments have been addressed

**********

2. Is the manuscript technically sound, and do the data support the conclusions?

Reviewer #1: Yes

Reviewer #2: Yes

**********

3. Has the statistical analysis been performed appropriately and rigorously?

Reviewer #1: Yes

Reviewer #2: Yes

**********

4. Have the authors made all data underlying the findings in their manuscript fully available?

The PLOS Data policy

Reviewer #1: Yes

Reviewer #2: Yes

**********

5. Is the manuscript presented in an intelligible fashion and written in standard English?

Reviewer #1: Yes

Reviewer #2: Yes

**********

Reviewer #1: (No Response)

Reviewer #2: The manuscript has been substantially improved and the reviewers’ comments have been appropriately addressed. One minor reporting issue remains: please explicitly report the number of participants included in the final multivariable logistic regression model, as previously requested. Following this minor clarification, the manuscript can be accepted without further review.

**********

what does this mean?). If published, this will include your full peer review and any attached files.

If you choose “no”, your identity will remain anonymous but your review may still be made public.

**Do you want your identity to be public for this peer review?**  For information about this choice, including consent withdrawal, please see our Privacy Policy

Reviewer #1: No

Reviewer #2: **Yes:**  OZLEM YILMAZ

**********

# Editor report: Acceptance letter

**DOI:** 10.1371/journal.pone.0361113.r004

**Authors:** Myadagmaa Jaalkhorol

PONE-D-26-22047R1

PLOS One

Dear Dr. Ida,

I'm pleased to inform you that your manuscript has been deemed suitable for publication in PLOS One. Congratulations! Your manuscript is now being handed over to our production team.

At this stage, our production department will prepare your paper for publication. This includes ensuring the following:

* All references, tables, and figures are properly cited

* All relevant supporting information is included in the manuscript submission,

* There are no issues that prevent the paper from being properly typeset

You will receive further instructions from the production team, including instructions on how to review your proof when it is ready. Please keep in mind that we are working through a large volume of accepted articles, so please give us a few days to review your paper and let you know the next and final steps.

Lastly, if your institution or institutions have a press office, please let them know about your upcoming paper now to help maximize its impact. If they'll be preparing press materials, please inform our press team within the next 48 hours. Your manuscript will remain under strict press embargo until 2 pm Eastern Time on the date of publication. For more information, please contact onepress@plos.org.

You will receive an invoice from PLOS for your publication fee after your manuscript has reached the completed accept phase. If you receive an email requesting payment before acceptance or for any other service, this may be a phishing scheme. Learn how to identify phishing emails and protect your accounts at https://explore.plos.org/phishing.

If we can help with anything else, please email us at customercare@plos.org.

Thank you for submitting your work to PLOS One and supporting open access.

Kind regards,

PLOS One Editorial Office Staff

on behalf of

Dr. Myadagmaa Jaalkhorol

Academic Editor

PLOS One
